# Correction: Porphyrin overdrive rewires cancer cell metabolism

**DOI:** 10.26508/lsa.202402816

**Published:** 2024-05-20

**Authors:** Swamy R Adapa, Gregory A Hunter, Narmin E Amin, Christopher Marinescu, Andrew Borsky, Elizabeth M Sagatys, Said M Sebti, Gary W Reuther, Gloria C Ferreira, Rays HY Jiang

**Affiliations:** 1 USF Genomics Program, Center for Global Health and Infectious Diseases, College of Public Health, University of South Florida, Tampa, FL, USA; 2 Department of Molecular Medicine, Morsani College of Medicine, University of South Florida, Tampa, FL, USA; 3 Department of Molecular Oncology, H. Lee Moffitt Cancer Center and Research Institute, Tampa, FL, USA; 4 Department of Pathology, H. Lee Moffitt Cancer Center and Research Institute, Tampa, FL, USA; 5 Department of Pharmacology and Toxicology, Massey Cancer Center, Virginia Commonwealth University, Richmond, VA, USA; 6 Department of Chemistry, College of Arts and Sciences, University of South Florida, Tampa, FL, USA; 7 Global and Planetary Health, College of Public Health, University of South Florida, Tampa, FL, USA

## Abstract

Cancer cells exhibit a metabolic phenotype termed “porphyrin overdrive,” characterized by dysregulated heme metabolic pathways for intermediate accumulation. This rewiring is cancer-essential and cancer-specific. Targeting this vulnerability with a “bait-and-kill” strategy shows promise in eradicating malignant cells.

Article: Adapa SR, Hunter GA, Amin NE, Marinescu C, Borsky A, Sagatys EM, Sebti SM, Reuther GW, Ferreira GC, Jiang RHY (2024 April 7) Porphyrin overdrive rewires cancer cell metabolism. Life Science Alliance 7(7): e202302547. doi: 10.26508/lsa.202302547. PMID: 38649187.

Upon reviewing our supplemental data, we noticed an assembly error in Fig S8, specifically in the lower panel. This panel was intended to demonstrate that only the parental strain K562 WT underwent additional staining for confirmation. We have updated this panel, including a magnified circle, to accurately depict this information.

**Figure S8.**
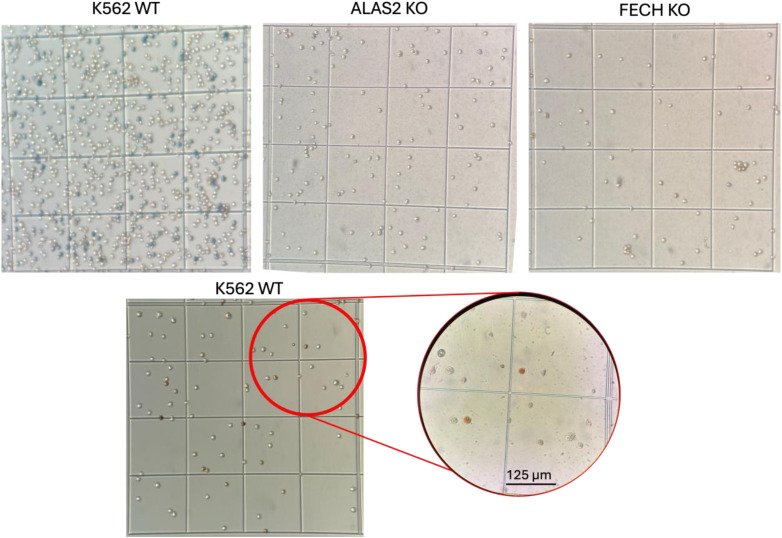
Erythroid differentiation in vitro erythropoiesis assays. The parent K562 cell line readily undergoes differentiation at 48 h post-induction in vitro. In contrast, neither the ALAS2 KO nor the FECH KO mutants show signs of differentiation (parent line versus mutants, *P* < 0.0001). Cell differentiation under 40 μM concentrations of hemin was assessed by staining cells with benzidine and o-dianisidine, where stained cells indicate hemoglobin accumulation and differentiation. The upper panel displays benzidine staining results for K562 WT, ALAS2 KO, and FECH KO, while the lower panel exhibits o-dianisidine staining for K562 WT. The magnified circle in the lower panel shows o-dianisidine-treated cells with varying degrees of staining.

## Supplementary Material

Reviewer comments

