# Supplementary material for: Correction: Porphyrin overdrive rewires cancer cell metabolism
Source: Life Sci Alliance. 2024 May 20;7(8):e202402816. doi: 10.26508/lsa.202402816 (PMC11106523; doi:10.26508/lsa.202402816)
Supplement: Reviewer comments [file LSA-2024-02816_review_history.pdf]

Life Science Alliance

Correction: Porphyrin overdrive rewires cancer cell metabolism

Swamy Adapa, Gregory Hunter, Narmin Amin, Christopher Marinescu, Andrew Borsky, Elizabeth Sagatys, Said Sebti, Gary Reuther, Gloria Ferreira, and Rays Jiang
DOI: https://doi.org/10.26508/lsa.202402816

Corresponding author(s): Rays Jiang, University of South Florida

Review Timeline:

Submission Date:	2024-05-10
Accepted:	2024-05-14

Scientific Editor: Eric Sawey, PhD

Transaction Report:

(Note: With the exception of the correction of typographical or spelling errors that could be a source of ambiguity, letters and reports are not edited. The original formatting of letters and referee reports may not be reflected in this compilation.)

May 14, 2024

RE: Life Science Alliance Manuscript #LSA-2024-02816

Dr. Rays H.Y. Jiang
University of South Florida
Global Health
3720 Spectrum Blvd
Tampa, FL 33612

Dear Dr. Jiang,

Thank you for submitting your Correction entitled "Correction: Porphyrin overdrive rewires cancer cell metabolism".

IMPORTANT: If you will be unreachable at any time, please provide us with the email address of an alternate author. Failure to respond to routine queries may lead to unavoidable delays in publication.

You can contact the journal office with any questions, contact@life-science-alliance.org

Sincerely,

Eric Sawey, PhD
Executive Editor
Life Science Alliance
http://www.lsajournal.org

May 14, 2024

RE: Life Science Alliance Manuscript #LSA-2024-02816

Dr. Rays H.Y. Jiang
University of South Florida
Global Health
3720 Spectrum Blvd
Tampa, FL 33612

Dear Dr. Jiang,

Thank you for submitting your Correction entitled "Correction: Porphyrin overdrive rewires cancer cell metabolism".

IMPORTANT: If you will be unreachable at any time, please provide us with the email address of an alternate author. Failure to respond to routine queries may lead to unavoidable delays in publication.

You can contact the journal office with any questions, contact@life-science-alliance.org

Sincerely,

Eric Sawey, PhD
Executive Editor
Life Science Alliance
http://www.lsajournal.org
